# Questionable Research Practices and Misconduct Among Norwegian Researchers

**DOI:** 10.1007/s11948-021-00351-4

**Published:** 2021-12-21

**Authors:** Matthias Kaiser, Laura Drivdal, Johs Hjellbrekke, Helene Ingierd, Ole Bjørn Rekdal

**Affiliations:** 1 Center for the Study of the Sciences and Humanities, University of Bergen, Bergen, Norway; 2 Department of Sociology, University of Bergen, Bergen, Norway; 3 The National Committees for Research Ethics in Norway, Oslo, Norway; 4 Department of Welfare and Participation, Western Norway University of Applied Sciences, Bergen, Norway

**Keywords:** Research integrity, Research misconduct, Questionable research practices (QRPs), Falsification, fabrication plagiarism (FFP)

## Abstract

This article presents results from the national survey conducted in 2018 for the project *Research Integrity in Norway* (RINO). A total of 31,206 questionnaires were sent out to Norwegian researchers by e-mail, and 7291 responses were obtained. In this paper, we analyse the survey data to determine attitudes towards and the prevalence of fabrication, falsification and plagiarism (FFP) and contrast this with attitudes towards and the prevalence of the more questionable research practices (QRPs) surveyed. Our results show a relatively low percentage of self-reported FFPs (0.2–0.3%), while the number of researchers who report having committed one of the QRPs during the last three years reached a troublesome 40%. The article also presents a ranking of the perceived severity of FFP and QRPs among Norwegian researchers. Overall, there is a widespread normative consensus, where FFP is considered more troublesome than QRPs.

## Introduction and background

Research integrity and breaches of research integrity have gained increased attention the last two decades. New forms of problematic conduct are also arising. Many countries have adopted research integrity guidelines, but these differ significantly between countries—even within Europe (Godecharle et al., 2014). Some countries have adopted laws to regulate ethics in research, among them Norway. A new Norwegian law, the Act concerning the organisation of work on ethics and integrity in research (Research Ethics Act), superseding the previous Act of 2006, was passed in 2017, delegating more responsibility to higher education and research institutions. This seems to be a general trend in Europe (see e.g. ALLEA, 2017; Forsberg et al., 2018; Science Europe 2016). However, in order to devise effective mechanisms to address research integrity and misconduct, more knowledge is needed about the prevalence, attitudes and possible drivers of misconduct. In reaction to the public consultative hearings leading to the new Norwegian Research Ethics Act, and in relation to international research on research integrity, the project Research Integrity in Norway (RINO) was initiated in 2016. A main aim of the project was to provide empirically-based knowledge on attitudes towards and the prevalence of misconduct and questionable research practices among Norwegian researchers. While some studies have investigated research integrity and dishonesty among Norwegian biomedical PhD students (Hofmann et al., 2013, 2015; Holm & Hofmann, 2017), the RINO project covered all disciplines and academic ranks, from research assistant to professor emeritus. Previously, a national study on scientific integrity had been carried out in 1997 (National Research Ethics Committees, 1997), but the sample of Norwegian researchers was much smaller (N = 456), and the response rate of (39%) may therefore have provided less reliable data.

In this paper, we will analyse the data produced by the RINO survey on the prevalence and attitudes towards misconduct and questionable research practices. We ask the following questions:

1. What are the attitudes towards and the prevalence of FFP and QRPs among Norwegian researchers?
2. How do Norwegian researchers rank the severity of different forms of FFP and QRPs?

### Research Integrity and Misconduct

In recent decades, there has been an increased focus on both the scale and the possible consequences of breaches of research integrity (Godecharle et al., 2014; Kaiser, 2014; Science Europe 2016; Shaw, 2019; Steneck, 2006). Benessia et al. (2016) suggest that scientific research seems less and less prepared for delivering the quality that is expected. Research ethics and integrity are not only important for upholding trust, but also for the overall quality of scientific output. In the literature on research integrity and misconduct, central concepts such as research integrity and research ethics are often conflated (Shaw, 2019; Steneck, 2006). Some use research ethics more narrowly about questions concerning research participants or groups affected by the research. In Norway however, research ethics is mainly used as an umbrella term, which includes *integrity* as one of the main features of the general principles of research ethics.^1^ In line with this, *research integrity* here refers more specifically to certain norms and principles that constitute good scientific practice, related to the quest for reliable knowledge, and norms and principles that regulate the research community. To the extent that scientific integrity is compromised, the resulting quality of science is also compromised. Therefore, it is of vital importance to have empirical data that can indicate the prevalence of breaches of integrity in the scientific community.

Research misconduct, is characterised by the lack of adherence to certain scientific norms. There is no uniform internationally recognised definition of research misconduct. According to Norwegian legislation, “scientific misconduct”^2^ means falsification, fabrication, plagiarism and other serious violations of recognised research ethics norms that have been committed intentionally or with gross negligence in planning, conducting or reporting research (Sect. 8 of the Norwegian Research Ethics Act of 2017). Usually, a distinction is made between falsification, fabrication and plagiarism (FFP) on the one hand, and questionable research practices (QRPs) on the other. While FFP comprises more or less clear categories,^3^ QRPs are less exhaustively elucidated. For example, while Bouter et al. (2016) list 60 different QRPs, the OECD (2008) lists 19 QRPs and emphasises that the current dynamics of research and its new methods (big data, digitalisation of data, etc.) may further extend such a list. Therefore, different studies often focus on different kinds of QRPs, and while some include a large list (such as the Bouter et al., 2016 survey), others focus on specific groups of QRPs such as citation and authorship manipulation (Fong & Wilhite, 2017; Smith et al., 2020). Further, it is noted that characterising some practices as merely *questionable* may seriously underestimate their severity in many individual cases (Shaw, 2019). It has been argued that FFP, due to its low prevalence in reality, is the lesser problem of integrity, while QRPs are still under-explored and their precise boundaries and contents are in flux (Bouter et al., 2016; Martinson et al., 2005; Shaw, 2019).

Internationally, research on misconduct—and especially on QRPs—has expanded (Bouter et al., 2016; Fanelli, 2009; Gopalakrishna et al., 2021; Haven et al., 2019; Martinson et al., 2005; Xie et al., 2021). A meta-study based on 21 international studies suggests that the prevalence of both FFP and QRPs generally is roughly of the same magnitude across national borders and disciplines (Fanelli, 2009). Fanelli (2009) found that close to 1.06% of the researchers admitted to FF, and 9.54%admitted to QRPs (p. 6). In a more recent meta-analysis, Xie et al. (2021) found a prevalence of 2.9% in self-reported FFP, and 12.5% in regard to QRPs. These are relatively high numbers, and if this trend continues or even increases, the utility and reputation of the scientific enterprise will be significantly damaged. Thus, efforts are increasingly being made to shape and enforce research integrity norms, guidelines and policies. However, guidelines for research integrity and ethics vary between countries (Aubert Bonn et al., 2017; Godecharle et al., 2013). Further, studies on attitudes towards and/or the prevalence of misconduct in different national contexts, such as Croatia (Pupovac et al., 2017), the Middle East (Felaefel et al., 2018) and Spain (Feenstra et al., 2021), indicate some differences. A recent pre-published study from the Netherlands found a surprisingly high rate of FF, with 4.3% and 4.2% respectively, and a high number of respondents (51.3%) who frequently engaged in at least one QRP (Gopalakrishna et al., 2021). One difference to earlier studies is the use of a randomised response technique in determining FF, aiming at excluding response and refusal bias. Similarly, their specification of possible QRPs includes practices that are not typically included in other studies (as e.g. “insufficient supervision or mentoring of junior co-workers” or “inadequate note taking of the research process”; ibid. p. 3). As the data sample and the questionnaire design in these studies vary substantially, they are not directly comparable, and one cannot draw reliable conclusions on historic development, national differences or other background characteristics. Assuming that national strategies could have an impact on research practices is, however, still viable as a working hypothesis.

In Norway, several national efforts have been initiated to promote research ethics and integrity. Three national committees for research ethics were established in 1990: one for science and technology, industry, agriculture and fishery research (NENT); one for medical and psychological research (NEM); and one for research in the social sciences and humanities (NESH). The Norwegian National Research Ethics Committees provide research ethics guidelines and offer advice and guidance to individual researchers and research institutions. Courses in the theory of science combined with ethical issues in science, are a mandatory part of a Norwegian PhD education, and research institutions are also required to offer such education to all employees and all who take part in research. With experiences from national and institutional integrity committees, the RINO project team initially anticipated that occurrences of FFP would be rare, while some occurrences of QRPs were to be expected.

Another topic that is underexplored in the literature is how different QRPs are assessed by researchers. An important contribution here is Bouter et al. (2016), who surveyed 60 different practices and found that respondents were more concerned about QRPs than FFP, particularly regarding the possible consequences of the high rates of QRPs (or “sloppy science”). However, the survey respondents were attendees at a conference on research integrity, and the authors noted that it remains to be assessed how common these behaviours really are and whether the rankings reflect their actual gravity. In our survey, as described below, the respondents are researchers from all disciplines and academic ranks.

## Methods and Data

The RINO project, which ran from 2016 to 2019, consisted of two empirical parts: a quantitative survey among Norwegian researchers, and a qualitative part with interviews and focus groups. In this article, we will analyse the main parts of the data from the survey on attitudes towards and the prevalence of FFP and QRPs, applying a univariate analysis.

### The Survey

The survey questionnaire^4^ was drafted by the RINO working group, inspired by similar national and international surveys (Anderson, 1996; Bouter et al., 2016; National Research Ethics Committees, 1997; Fanelli, 2009; Martinson et al., 2005). It was decided not to copy these surveys directly, but to rather establish a simple and short survey that could provide a large amount of data, as the main aim of the RINO project was to provide insights into the conditions in Norway and thus provide input to national plans and regulations. Thus, questions about FFP and nine QRPs were included. In order to increase the response rate, it was important to keep the survey relatively short. The selected QRPs were to be relevant for all disciplines and academic ranks. QRPs related to research methods (e.g. lack of informed consent), which differ significantly between disciplines, were therefore excluded. The questionnaire also included an open comments field. Three types of questions were asked for FFP and for the nine QRPs: (1) the respondents’ attitude to the practice; (2) the respondents’ knowledge about the frequency of this practice among their immediate colleagues during the last three years; and (3) the respondents’ own engagement in this practice during the last three years.

Prior to sending out the questionnaire, a quality control check was performed through discussions with the project’s reference group and a pilot with volunteers. The authors are aware of the many pitfalls in analyses of scientific misconduct, as e.g. possible bias introduced in the way survey questions are phrased and selected, with QRPs as the most controversial practices (Zuckerman, 2020, Kaiser, 2014). Quality assurance of the questionnaire was therefore given a high priority. The project was granted approval by the Norwegian Centre for Research Data (NSD) for processing personal data. The personal data has since been deleted in accordance with the agreement, and it is not possible to identify individuals from the survey results.

## Results and Discussions

### Sample

The sample was compiled from publicly available e-mail lists at Norwegian universities, university colleges and research institutes. The overview of the institutions was taken from the Nordic Institute for Studies in Innovation, Research and Education (NIFU) and includes nearly the entire research community in Norway. There may be a few potential sources of error, such as double registration of researchers with more than one workplace, or underrepresentation, where a researcher for some reason is not included on the institution’s e-mail list. A very small number of institutions are not represented at all because of unavailability of e-mail addresses of their employees.

The questionnaire was e-mailed to 31,206 staff at the aforementioned types of institutions in three languages: Norwegian (Bokmål), New Norwegian (Nynorsk) and English.^5^ The respondents were asked to choose their preferred language when answering the questionnaire. We have not been able to detect any systematic differences in response profiles that can be attributed to a language factor. The first batch was sent on Friday, 19 January 2018. Two reminders were sent to every e-mail address, which included a link to the questionnaire. Wednesday, 1 March was set as the final date for data collection, i.e. a timeframe of almost seven weeks. At this point, a total of 7947 researchers had responded to all or parts of the survey, which implies a response rate of 25.5%. The number that completed the entire questionnaire was 7291—a response rate of 23.4% (Table 1). The number of respondents was larger than in many other international surveys on research misconduct and QRPs, with for instance Gopalakrishna et al., 2021 reporting a response rate of 21.2% and 6813 completed responses. One notable exception is a survey on authorship and citation manipulation, which analysed data from over 12,000 responses to a series of surveys sent to more than 110,000 scholars (Fong & Wilhite, 2017).

**Table 1:** Survey characteristics

Total sample size	31,206	100%
Responses	7947	25.5%
Complete responses	7291	23.4%

### Sample Bias

The distribution in the RINO sample from 2018 is comparable to the distribution in the publicly available NIFU database for 2016, the official data on Norwegian research from NIFU (www.nifu.no) as regards four key variables: gender, job category, discipline and age.

The gender distribution in the RINO survey and in the NIFU database is identical: 48% women and 52% men. However, broken down into the various job categories, some biases emerge. The percentage of female professors is somewhat higher in our sample, and the proportion of female associate professors is somewhat lower than in the population. However, this is a very weak bias, which we do not believe should initiate corrective weighting.

Research positions like professor and associate professor were overrepresented and positions such as e.g. lecturers and senior lecturers were correspondingly underrepresented in the final answers. Given the topic of the study—research integrity—this result was to be expected. We see this biased distribution as a result of self-selection.

In terms of disciplines, mathematics/natural sciences are overrepresented by 4.6%, while social sciences are underrepresented by 6.3% and medicine by 3.6% in the sample as a whole. However, among university staff, medicine is overrepresented by 6%. The greatest disparity is in the university college component of the sample, where the social sciences are more strongly underrepresented and the humanities are correspondingly overrepresented.

Finally, the response rate is somewhat higher among older than younger respondents, while the disparity between the population and the distribution in the university component of the sample is minimal.

Based on an overall assessment, the research group decided against weighting the sample based on any of these variables or combination of variables, since this could result in creating new biases. Therefore, in the analyses that follow, the results presented are all unweighted.

### FFP

The results for FFP are given in the tables below:

Let us briefly summarise these results. Firstly, from Table 2, it is quite obvious that Norwegian researchers have very little tolerance of FFP practices, they more or less uniformly condemn fabrication and falsification of material, while there is a bit more uncertainty about plagiarism. Note, however, that among the total respondents (> 7200), there were still roughly a bit more than 100 respondents who did not see larger problems with each of these forms of scientific practice. And roughly 10% were slightly in doubt about plagiarism.

**Table 2:** Attitudes towards practices: fabrication, falsification and plagiarism

	To fabricate (invent) data/material	To falsify data/material	To present other people’s work (ideas, material, text) as your own by excluding a reference to the original source (plagiarism)
This is not problematic at all	0.9%	0.9%	0.7%
This is somewhat problematic	0.6%	0.4%	0.8%
This is quite problematic	1.2%	0.7%	8.4%
This is very problematic	97.3%	97.9%	90.1%
Total	100% (N = 7241)	100% (N = 7239)	100% (N = 7246)

Secondly, when it comes to self-reporting of FF practices (Table 3), our numbers deviate from the numbers presented in the meta-analysis by Xie et al. (2021), where 2.9% respondents admitted to FFP, and the recent study by Gopalakrishna et al. (2021) where 4.3% and 4.2%, respectively, admitted to one form of FF. As mentioned earlier, these numbers are not directly comparable, while one still has the impression that the numbers in our study are relatively small, 0.2% and 0.3%, respectively, for each FF practice. Whether this is due to different survey techniques or it captures different research cultures with varying practices, is open to interpretation.

**Table 3:** Self-admission of practices: fabrication, falsification and plagiarism

	Have you yourself engaged in this type of practice in the last three years?		
	To fabricate (invent) data/materia^a^	To falsify data/material^b^	To present other people’s work (ideas, material, text) as your own by excluding a reference to the original source (plagiarism)^c^
No	99.8%	99.7%	99.5%
Yes, once	0.07%	0.2%	0.3%
Yes, a few times	0.04%	0.1%	0.2%
Yes, several times	0.06%	0%	0.01%
Total	100% (N = 7129)	100% (N = 7127)	100% (N = 7181)

^a^A total of 12 respondents report having fabricated data on at least one occasion

^b^A total of 19 respondents report having falsified data/material on at least one occasion

^c^A total of 35 respondents report having plagiarised a work on at least one occasion

With regard to plagiarism, we note that > 0.5% admit having done it at least once during the last three years. However, this result should also be seen in conjunction with the next table, in particular about copying citations.

### QRPs

Our survey contained nine different practices that can be termed QRPs. Here, we present the univariate analysis of attitudes towards and self-reported admissions of these nine practices:

The two tables present some variances in attitudes towards the practices and the self-admission of the practices. In the following, we will discuss the differences between the attitudes and self-admission of four of the QRPs in these tables.

#### Copying References

Concerning the first practice: to create the impression of having consulted a source by copying other’s citations, 22.2% (Table 4) of the respondents report a relatively liberal attitude to this, classifying the practice as “somewhat problematic” or even “not problematic at all”. Roughly 20% (Table 5) report having done it at least once in the last three years. The way this particular question was formulated implies a practice that has been termed “citation plagiarism” (e.g. Serenko et al., 2021), an academic shortcut that may potentially cause misleading or false information to be reproduced in the literature, contributing in some cases to the spread of “academic urban legends” (Rekdal 2014).

**Table 4:** Attitudes towards questionable practices

	To create the impression of having consulted a source by copying others’ citations	To accept, mandate or allocate authorship based on criteria other than significant contribution to a scientific work (gift authorship)	To deny or omit authorship despite significant contribution to a scientific work	To break up or segment study results into two or more publications to boost your publication credits, at the expense of scientific quality (salami slicing)	To change the design, methodology and/or results of a study in response to pressure from stakeholders or funding sources	To use research data/material when its ownership is contested	To refrain from informing end-users and decision-makers about significant limitations and/or uncertainties in the data material, analysis and/or conclusion	To refrain from reporting (whistle blowing) serious breaches of research ethical guidelines	To include irrelevant or unnecessary references in a publication in order to increase the citation frequency of a colleague, a research environment or a journal
This is not problematic at all	1.2%	1.7%	1.6%	4.3%	1.1%	1.6%	0.8%	1.2%	2.0%
This is somewhat problematic	21.0%	12.4%	3.7%	31.3%	5.3%	11.9%	5.0%	5.2%	26.7%
This is quite problematic	47.1%	34.1%	14.6%	43.4%	22.5%	42.1%	31.6%	30.5%	45.4%
This is very problematic	30.7%	51.7%	80.1%	21.0%	71.1%	44.5%	62.6%	63.1%	25.9%
Total	100% (N = 7236)	100% (N = 7240)	100% (N = 7243)	100% (N = 7226)	100% (N = 7223)	100% (N = 7181)	100% (N = 7266)	100% (N = 7224)	100% (N = 7228)

**Table 5:** Self-admission of questionable research practices during the last three years

	To create the impression of having consulted a source by copying others’ citations	To accept, mandate or allocate authorship based on criteria other than significant contribution to a scientific work (gift authorship)	To deny or omit authorship despite significant contribution to a scientific work	To break up or segment study results into two or more publications to boost your publication credits, at the expense of scientific quality (salami slicing)	To change the design, methodology and/or results of a study in response to pressure from stakeholders or funding sources	To use research data/material when its ownership is contested	To refrain from informing end-users and decision-makers about significant limitation and/or uncertainties in the data material, analysis and/or conclusion	To refrain from reporting (whistle blowing) serious breaches of research ethical guidelines	To include irrelevant or unnecessary references in a publication in order to increase the citation frequency of a colleague, a research environment or a journal
No	79.3%	88.7%	98.2%	91.6%	95.6%	98.9%	97.5%	95.3%	87.5%
Yes, once	6.4%	5.9%	1.3%	4.1%	2.6%	0.8%	1.2%	2.9%	5.6%
Yes, a few times	13.7%	4.7%	0.5%	4.1%	1.7%	0.3%	1.2%	1.7%	6.6%
Yes, several times	0.6%	0.4%	0.04%	0.2%	0.1%	0.04%	0.1%	0.1%	0.3%
Total	100% (N = 7154)	100% (N = 7116)	100% (N = 7123)	100% (N = 7111)	100% (N = 7134)	100% (N = 6983)	100% (N = 6953)	100% (N = 7101)	100% (N = 7154)

#### Gift Authorship

Authorship issues are known to be contentious in the scientific community, and guidelines have been issued to clarify the rightful claim to be an author or co-author of a scientific paper. These guidelines are typically modelled on the Vancouver recommendations, demanding significant contributions to various stages in the production of the research and the resulting papers. The guidelines have been endorsed by COPE (Committee on Publication Ethics). However, despite the fact that many scientific publishers are members of COPE, experience indicates that compliance with these guidelines and their practical implementation is not uniform but varies between age groups, disciplines and countries. It is, therefore, of interest to see how the topic of gift authorship is viewed and practised among Norwegian researchers, in particular because the national Norwegian ethical guidelines are quite clear on this point (NENT, 2016, Guideline 5; NESH, 2016, Guideline 25).

The complexity of the issue is reflected in the results related to attitudes towards gift authorship. About 14% of the respondent’s regard this as not problematic or only somewhat problematic. Whether this should be considered a high percentage is up for discussion, but it certainly indicates that the whole issue is seen as more complex and not so straightforward as normally dealt with in the ethical guidelines. Given this variation in the views on gift authorship, it is then not surprising to find that roughly 11% of the respondents admit to having been involved in gift authorships in the last three years.

We surmise that the issue of authorship assignments becomes more important with increased expectations and rewards for publications based on simple quantitative measures, like the Hirsch index or similar. “Pressure” is the one term that was mentioned most frequently in the open comments section of the survey. Increased competition among researchers and research groups for funding and positions was mentioned in this connection.

Furthermore, the issue of gift authorship reflects internal power structures within the scientific community. Theoretically, motivations to assign gift authorship may be twofold. Firstly, the inclusion of the name of a highly regarded researcher will increase the likelihood of the paper having a substantial impact and being cited within the relevant community. Secondly, a supervisor or other senior, powerful member of the research group may expect to be mentioned as co-author. For many, in particular younger researchers, it is difficult to deny this request without negative repercussions. Both cases reflect the hierarchical structures within the research community and are reinforced by the reward system of funding and careers.

#### Salami Slicing

The practice of salami slicing, i.e. the breaking up of research findings into the maximum of “least publishable units” (Broad, 1981), follows the logic of the authorship issue discussed above: the higher the number of publications, the greater the rewards. Here, we note that more than 35% of the respondents do not find this practice problematic or only somewhat problematic, despite the formulation used in the question “at the expense of scientific quality”. There is, of course, a rationale for this viewpoint, namely that the very content of the scientific communication is assumedly not essentially changed whether published in one piece or in several. The reason for regarding the practice as ethically problematic is not anchored in the truth content, but in the ease of reception in the scientific community and in chopping up research which was designed to form a unit. A further reason is that it disproportionally distorts the work effort behind the publications: “hard numbers on a curriculum vitae no longer necessarily add up to hard work” (Broad, 1981, 1137).

Given the high rate of acceptance of salami slicing, it is perhaps a bit surprising to find that only about 8% admit to having engaged in this practice. Whether this is a true reflection of the researchers’ reality might, of course, be questioned. There is always a well-known bias in assigning ethically problematic behaviour to others as opposed to assigning the behaviour to oneself. We tend to be more lenient with ourselves. Thus, researchers might find good reasons to segment their own research results, even though outsiders might question this and would have expected a more comprehensive publication in a single piece.

#### Response to External Pressure

One feature of the more recent realities of research is the closer interaction with external funders and stakeholders in a research project. Often, this interaction is a contractual requirement for the research funds. The interaction and often collaboration with these non-scientific bodies and actors could potentially raise the level of conflict, as they may enter the project with differing objectives and interests. This intermingling of interests and objectives has led to public concern that scientific research could become instrumentalised for powerful interests and that reported results could be biased, thus compromising full veracity.^6^ One mechanism would be for funders or stakeholders to exert pressure on the scientists to change the study design, methodology or the published results of the study, according to their interests. There is a clear perception among most sectors of society that this would impede the freedom of science and diminish confidence in its operations. Yet, there are frequently reports that this is indeed happening (Ingierd, et al., 2019; National Research Ethics Committees, 2003), in spite of some institutional measures—for instance standard contracts for commissioned research – to prevent this practice.

The data (Table 5) shows that 94% of the respondents find this unacceptable; correspondingly, 95% (Table 6) report not having experienced such pressure. Whether or not this result should be considered reassuring depends on i.e. the percentage of respondents who regularly receive external funding and are principal investigators in such research projects. Since 78% of our respondents were from universities or from university colleges, and only 22% from research institutes, the average dependency on external funding in Norway may have been low.

**Table 6:** Reported own, conducted questionable practices (N = 7223)

Performed questionable practices	Frequency	Percent	Cumulative percent
none	4373	60.5	60.5
one	1766	24.4	84.9
two	741	10.3	95.2
three	231	3.2	98.5
four	77	1.1	99.6
five or more	35	0.4	100
total	7223	100	

Respondents with missing values ​​for more than five variables have been omitted from the analysis. For the remaining respondents, missing values are coded with “No”. Therefore, the percentage in the “None” row may be marginally lower than the table indicates

## Engagement with One of the QRPs

In Table 6, we have summarised the values of the respondents across the nine QRP variables with regard to their own engagement with them:

We note that roughly 40% of our respondents admit to having engaged in at least one QRP in the last three years. This is a significantly higher number than we expected from the literature (e.g. Fanelli, 2009).

## Ranking of FFP and QRPs

One of our guiding research questions was the ranking of the perceived severity of different forms of FFP and QRPs. The results are summarised in the following table.

What emerges clearly in Table 7 is that there is a perceived hierarchy among different forms of FFP and QRPs in the assessment of the respondents. In general, one may conclude from the results that there is a widespread normative agreement among Norwegian researchers of good and bad research practices, with only two or three QRP practices (out of our list of nine) slightly disputed. With this broad normative consensus, one would expect a relatively unproblematic research practice with regard to the nine forms of QRP. However, the self-reported data (Table 6) reveals that 40% of the respondents have engaged in one or more forms of QRP during the last three years.

**Table 7:** Ranking of practices by the percentage of respondents who answered “Very problematic”

	Percentage, “Very Problematic” (%)
Falsify data	97.9
Fabricate data	97.3
Plagiarism	90.1
Deny authorship despite significant contribution	80.1
Change the design, methodology and/or results of a study in response to pressure	71.1
Refrain from whistle blowing	63.1
Refrain from informing about limitations and/or uncertainties	62.6
Gift authorship	51.7
Use data when its ownership is contested	44.5
Copying others’ citations	30.7
Include irrelevant references to increase citation frequency	25.9
Salami slicing	21.0

## Conclusion

The RINO study delivered robust numbers on FFP and QRPs among Norwegian researchers. With regard to fabrication and falsification, one noteworthy result is that the percentage of researchers who admitted to engaging in one of these practices, is somewhat lower than reported in other international studies or meta-analyses. However, since we are dealing with the low end of a large spectrum, namely the range between 0.2% and 1.06%, and since there is significant uncertainty regarding the reliability of the data, one may be inclined to dismiss the difference as less problematic. This may be a big mistake, though. We consider this a significant potential danger to the integrity and trustworthiness of science.

Given this data, and given the high number of those who admitted to engaging in at least one form of QRP during the last three years, we are hesitant regarding the assumed positive impact of the Norwegian Research Ethics Act or other institutional measures to improve ethics in research. It seems there has been no clear improvement when compared to earlier studies (Hofmann et al., 2013, 2015; National Research Ethics Committees, 1997). When 40% of our respondents report having engaged in one or more forms of QRP during the last three years, in spite of increased institutional measures to raise awareness of research ethics in the Norwegian scientific community and to address allegations of misconduct, the conclusion must be that we have not reached the end of the discussion.

Another finding is the complex picture in relation to the attitudes towards QRPs. It seems to us that a lot of discussion and clarification is needed in order to improve the ability of researchers to assess the issues. However, more detailed normative clarifications of some practices may also be needed. The next question must then be what kind of measure one would need to remedy this. In our view, it seems that legal reforms will achieve little in this regard. The problem may be deeply entrenched in scientific culture and its institutions.

## Footnotes

1 https://www.forskningsetikk.no/en/guidelines/general-guidelines/

2 The Norwegian term is “vitenskapelig uredelighet”.

3 Some definitions in certain national guidelines demand positive proof of intentionality behind the act, thus excluding mere sloppiness, and some definitions add phrases like e.g. “and other serious breaches of scientific norms”. Both Zuckerman (2020) and Kaiser (2014) discuss how the development of unified definitions of FFP was influenced by historic contingencies, and they thus introduce a certain amount of judgemental bias.

4 The questionnaire is presented in “Appendix 1”.

5 The research group consists of members with *Bokmål* as their native language, and members with *Nynorsk* as their native language. In this sense, the research group is bilingual. When the questionnaire was drafted, all members actively took part in the formulation and verification of the wording in the questions. Native *English* speakers who also speak and write Norwegian fluently were asked to verify and correct the translation into English.

6 The important term here is “pressure”. In the Norwegian research community, there has been a lot of attention on commissioned research and where the border lies between acceptable interaction with funders and unacceptable pressure. It is clearly stated in national guidelines and standard contracts that the final responsibility for scientific quality always rests with the researcher, and that funders may suggest improvements, but may not exert pressure through specific research designs. Cf. Ingierd et al. (2019) and National Research Ethics Committees (2003).
